# Supplementary material for: Incidence of Cholangitis and Sepsis Associated with Percutaneous Transhepatic Cholangiography in Pediatric Liver Transplant Recipients
Source: Antibiotics (Basel). 2021 Mar 10;10(3):282. doi: 10.3390/antibiotics10030282 (PMC8001276; doi:10.3390/antibiotics10030282)
Supplement: Supplementary file 1 [file antibiotics-10-00282-s001.pdf]

antibiotics

MDPI

Article

Incidence of Cholangitis and Sepsis Associated with Percutaneous Transhepatic Cholangiography in Pediatric Liver Transplant Recipients

Naire Sansotta [1,*], Ester De Luca [2], Emanuele Nicastro [1], Alessandra Tebaldi [3], Alberto Ferrari [4] and Lorenzo D'Antiga [1]

1. Paediatric Hepatology, Gastroenterology and Transplantation, Hospital Papa Giovanni XXIII, Bergamo postal code, Italy; enicastro@asst-pg23.it (E.N.); ldantiga@asst-pg23.it (L.D.A.)
2. Department of Pediatrics, University of Milano Bicocca, Milan postal code, Italy; e.deluca@campus.unimib.it
3. Infectious Diseases Unit, Hospital Papa Giovanni XXIII Bergamo postal code, Italy; atebaldi@asst-pg23.it
4. FROM Research Foundation, Statistics, Hospital Papa Giovanni XXIII, Bergamo postal code, Italy; aferrari34@yahoo.com

* Correspondence: nsansotta@asst-pg23.it

Table S1. Antibiotic susceptibility pattern of germs isolated in blood and bile cultures.

**Antibiotic**	**Germs Isolated**								
**Susceptibility rate (%)**	*Enterococcus faecium* *N 15*	*Pseudomonas aeruginosa* *N 10*	*Escherichia coli* *N 5*	*Klebsiella pneumoniae* *N 4*	*Staphylococcus aureus* *N 2*	*Enterobacter aerogenes* *N 2*	*Enterococcus faecalis* *N 2*	*Enterobacter cloacae* *N 1*	*Acinetobacter baumanii* *N 1*
Ampicillin	1/15 (7%)	0/10 (0%)	0/4 (0%)	0/4 (0%)	0/2 (0%)	0/2 (0%)	2/2 (100%)	0/1 (0%)	0/1 (0%)
Ampicillin-sulbactam	–	0/10 (0%)	1/5 (20%)	0/4 (0%)	2/2 (100%)	0/2 (0%)	–	0/1 (0%)	0/1 (0%)
Penicillin G	0/15 (0%)	–	–	–	0/2 (0%)	–	0/2 (0%)	–	–
Piperacillin-Tazobactam	–	5/10 (50%)	3/5 (60%)	0/4 (0%)		0/2 (0%)	–	0/1 (0%)	–
Oxacyllin	–	–	–	–	2/2 (100%)	–	–	–	–
Cefotaxime	-	0/10 (0%)	0/5 (0%)	0/4 (0%)		0/2 (0%)	-	-	-
Cefuroxime	0/15 (0%)	–	–						
Gentamycin	0/3 (0%)	10/10 (100%)	3/5 (60%)	0/4 (0%)	2/2 (100%)	2/2 (100%)	2/2 (100%)	1/1 (100%)	1/1 (100%)
Cotrimoxazole	–	0/10 (0%)	2/5 (40%)	0/4 (0%)	2/2 (100%)	2/2 (100%)	–	1/1 (100%)	–
Ciprofloxacin	–	5/10 (50%)	2/5 (40%)	0/4 (0%)	–	1/2 (50%)	–	1/1 (100%)	0/1 (0%)
Amikacin	–	–	1/2 (50%)	4/4 (100%)	–	–	–	–	1/1 (100%)
Imipenem	0/15 (0%)	–	2/2 (100%)	2/4 (50%)	–	–	1/2 (50%)	–	–
Meropenem	–	–	3/3 (100%)	4/4 (100%)	–	2/2 (100%)	–	–	1/1 (100%)
Ertapenem	–	0/10 (0%)	2/2 (100%)	2/4 (50%)	–	1/2 (50%)	–	0/1 (0%)	0/1 (0%)
Cefepime	–	–	0/3 (0%)	0/4 (0%)	–	–	–	–	0/1 (0%)
Colistin	–	2/2 (100%)	1/1 (100%)	–	–	–	–	–	–
Ceftazidime	–	10/10 (100%)	–	0/4 (0%)	–	0/2 (0%)	–	0/1 (0%)	–
Tigecycline	2/3 (33%)	0/10 (0%)	1/1 (100%)	2/4 (50%)	–	–	–	–	–
Fosfomycin	–	–	1/1 (100%)	2/4 (50%)	–	–	1/2 (50%)	–	0/1 (0%)
Chloramphenicol	–	0/10 (0%)	–	–	–	–	–	–	0/1 (0%)
Doripenem	–	–	–	2/4 (50%)	–	–	–	–	–
Streptomycin	1/4 (25%)	–	–	–	–	–	2/2 (100%)	–	–
Erythromycin	0/15 (0%)	–	–	–	2/2 (100%)	–	0/2 (0%)	–	–
Clindamycin	0/15 (0%)	–	–	–	2/2 (100%)	–	0/2 (0%)	–	–
Vancomycin	12/15 (80%)	–	–	–	2/2 (100%)	–	2/2 (100%)	–	–
Linezolid	4/4 (100%)	–	–	–	–	–	1/2 (50%)	–	–
Levofloxacin	-	–	–	–	2/2 (100%)	–	–	–	–
Teicoplanin	1/2 (50%)	–	–	–	–	–	–	–	–
**VRE**	**3/15**	–	–	–	–		–	–	–
**ESBL**	–	–	**2/15**	**2/4**	–	**2/2**	–	**1/1**	–

**Carbapenemase Producing**	–	–	**1/15**	–	–	–	–	–	–

-: not tested, VRE: vancomycin-resistant Enterococcus sp.; ESBL: extended-spectrum beta-lactamase.
